# Effect of Refined Nursing on Wound Complications After Thoracoscopic Surgery for Lung Cancer

**DOI:** 10.1111/crj.70039

**Published:** 2025-01-20

**Authors:** Yanxia Dong, Guangqin Ma, Shuaishuai Wu, Shuling Zhang

**Affiliations:** ^1^ Cardiothoracic Surgery Department Dongying People's Hospital Dongying China; ^2^ Department of ENT Dongying People's Hospital Dongying China

**Keywords:** radical resection of lung cancer, refined nursing intervention, thoracoscopy, wound complications

## Abstract

**Introduction:**

Lung cancer thoracoscopic postoperative wound complications bring great pain and inconvenience to patients.

**Methods:**

To provide clinical nurses with a more scientific and effective nursing plan, this study evaluated the effect of refined nursing on wound complications after thoracoscopic surgery for lung cancer. Two‐hundred thirty patients undergoing thoracoscopic radical resection for lung cancer were randomly divided into two groups according to the random number table method. The study group received refined nursing intervention (115 cases), and the control group received routine nursing intervention (115 cases). The effects of the two groups' nursing modes on the wound complications (pleural effusion, incision infection, lung leakage, and wound bleeding) after thoracoscopic surgery for lung cancer were compared.

**Results:**

According to the study results, the study group experienced a shorter intraoperative blood loss, lower extubation time, shorter hospital stay, and lower wound complication rate than the control group (*p* < 0.05). Compared to the control group, the study group had significantly lower Hamilton Anxiety Scale (HAMA), Hamilton Depression Scale (HAMD) and visual analog scale (VAS) scores, and higher the short form 36 health survey questionnaire (SF‐36) scores (*p* < 0.05).

**Conclusion:**

Compared with the control group, the implementation of refined nursing intervention for patients with thoracoscopic radical resection of lung cancer has fewer postoperative wound complications and can improve patients' nursing satisfaction and quality of life, which is worthy of clinical promotion and application.

## Graphical abstract

In this study, different nursing modes were evaluated and compared in patients undergoing thoracoscopic radical lung cancer resection to determine which is more effective in managing postoperative wound complications. Compared with the control group, the implementation of refined nursing intervention for patients with thoracoscopic radical resection of lung cancer has fewer postoperative wound complications and can improve patients' nursing satisfaction and quality of life.

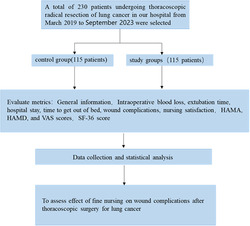

## 1 Introduction

Lung cancer is the most common type of cancer worldwide, and the therapeutic effect is not ideal, the survival time is short, and it is seriously harmful to people's health [1]. In recent years, the incidence of lung cancer in China has been increasing year by year, and the 5‐year survival rate is 8% to 15% [2, 3], which is a serious threat to the life and health of patients. For the treatment of early lung cancer, the first choice for clinical treatment of lung cancer is surgery [4], by removing cancerous tissue and infiltrating tissue to prolong the life cycle of patients and improve the survival rate of patients.

However, some studies have shown that for patients undergoing radical resection of lung cancer, the postoperative rehabilitation effect is poor because of the large surgical wound [5]. The process of postoperative rehabilitation may be affected by slow wound healing, pain and discomfort, slow recovery of respiratory function, and so on [6]. Furthermore, infections, bleeding, and respiratory problems are more likely to happen following surgery. Thoracoscopic radical resection of lung cancer is widely used in clinical practice because of its minimally invasive, less complications, and rapid recovery [7]. However, the operation of this kind of minimally invasive surgery is very demanding and requires high‐quality and systematic nursing [8]. It is of great significance to ensure the effect of operation and improve the prognosis of patients.

Refined nursing is one of the new nursing models advocated by modern nursing, which can optimize the nursing links and contents, ensure the smooth connection of all aspects of the operation, and provide refined and high‐quality nursing services for patients [9]. Refined nursing pays attention to the principle of “precision, refined, strict, and accurate.” According to the actual situation of the patients [10], a series of rigorous and accurate nursing interventions can be carried out to make the patients cooperate with the operation in the best state, so as to speed up postoperative recovery and reduce postoperative wound complications [11]. At present, there are few studies on the effect of refined nursing on postoperative wound complications in patients undergoing thoracoscopic radical resection of lung cancer [12]. Therefore, it is very important to understand the effect of refined nursing intervention on postoperative wound complications in patients undergoing thoracoscopic radical resection of lung cancer. We can improve the clinical treatment effect, reduce complications, and improve the quality of life of patients by preventing and treating postoperative wound complications.

The purpose of this study is to explore the effect of refined nursing on wound complications after thoracoscopic surgery for lung cancer, in order to make doctors and patients cooperate to improve the factors affecting wound complications, in order to provide reference for clinical treatment.

## 2 Data and Methods

### 2.1 General information

This study utilized a retrospective, unicentric design, involving 230 patients who underwent thoracoscopic surgery for lung cancer between March 2019 and September 2023, and patients were categorized into two groups based on different nursing regimens. and patients were categorized into two groups based on different nursing regimens. The study group was given refined nursing intervention (115 cases), and the control group was given routine nursing intervention (115 cases). Since this study was retrospective, no formal sample size calculation was done.

## 3 Inclusion and Exclusion Criteria

Inclusion criteria [13] (1) accorded with the relevant diagnostic criteria of lung cancer by imaging and laboratory examination; (2) met the relevant indications of radical resection of lung cancer; (3) received thoracoscopic radical resection of lung cancer; (4) adults over 18 years of age; and (5) no serious organic lesions: clear consciousness, volunteer to participate in this study.

Exclusion criteria included (1) severe liver and renal function damage; (2) severe mental abnormality; (3) blood coagulation dysfunction or severe anemia; and (4) unclear consciousness or inability to cooperate with this study.

### 3.1 Ethical Approval

Approval for this study was granted by the Dongying People's Hospital Research Ethics Committee, with all patients being fully informed and participating in the study voluntarily. The number is DPH‐20241109.

### 3.2 Nursing Methods

Control group: Routine nursing mode was adopted [14]: (1) itinerant nurses: carefully check patients' information with transfer nurses to establish deep venous access to help patients maintain healthy lateral position; assist anesthesiologists to perform operations such as anesthesia induction and endotracheal intubation; (2) instrument nurses: prepare various surgical items and cooperate with surgeons' incisions and puncture. In addition, it is necessary to assist the surgeon to operate the thoracoscope and flush and close the chest; and (3) closely observe the vital signs of the patient during the operation.

Study group: A refined nursing intervention was implemented based on the control group. Patients in both groups received nursing care for a duration of two weeks.

The specific measures are as follows [15]:

Preoperative care: Before surgery, nurses met with the patients to thoroughly record and analyze their surgical history and allergy history. During this process, we provided information about the main surgical procedures and the operating environment, using a combination of visual aids and text for health education. We guided patients in performing cardiopulmonary function exercises and abdominal breathing to enhance their preoperative physical preparation. Nurses actively communicated with the patients to assess their psychological status. If a patient's psychological condition was found to be unsatisfactory, timely psychological support and guidance were provided to help enhance the patient's confidence in their treatment.

Intraoperative care: During the surgery, calming music was played in the operating room, and roaming nurses continued to communicate with the patients to soothe their emotions and minimize equipment noise. According to the surgical safety checklist, the attending physician and the circulating nurse verified the patient's information and appropriately adjusted the patient's position after administering general anesthesia. Suitable soft padding was placed on the main pressure points of the patient, and the tightness of the restraint straps was adjusted to reduce skin exposure during the operation, thereby enhancing patient comfort. During catheterization after anesthesia, an appropriate amount of lidocaine cream was applied to the urinary catheter to avoid strong stimulation that could lead to restlessness during the recovery period. Fluids were prewarmed before infusion or chest irrigation, and care was taken to observe whether any drains or instruments were compressing the patient's skin during the procedure. The number of items used was checked postoperatively.

Postoperative care: After the surgery, blood was carefully cleaned from the patient, and assistance was provided to ensure the patient was neatly dressed. When transferring the patient, special attention was given to the chest tube to avoid any kinks, distortions, or detachments. Additionally, nurses explained to the patients the causes of postoperative incision pain and utilized distraction techniques and other approaches to help reduce discomfort. Moreover, nurses closely monitored for the occurrence of any postoperative complications, ensuring timely assessment and management.

### 3.3 Observation Index

The following indexes were observed before nursing and after 2 weeks of nursing in the two groups. The main results were as follows:

1. The amount of intraoperative blood loss, time of extubation, and time of hospitalization were compared between the two groups.
2. The incidence of postoperative wound complications (pleural effusion, incision infection, pulmonary air leakage, and bleeding wound) was recorded.
3. HAMA, HAMD, and VAS scores [16]: The HAMA scale consisted of 14 items; each item was scored on a 5‐point scale from 0 to 4, with higher scores indicating greater anxiety; the HAMD scale consisted of 17 items, each of which was scored on a hierarchical scale (1, 2, 3, 7, 8, 9, 10, 11, 15, and 17 scored on a scale of 0 to 4, 4, 5, 6, 12, 13, 14, and 16 were scored according to 0–2 points). The higher the score, the greater the degree of depression; the higher the VAS score, the greater the degree of pain.
4. Quality of life assessment [17]: SF‐36 score, total score 100 points, a higher final score indicates a better quality of life for the patient. There are eight items on the SF‐36 (general health, social function, physical function, emotional function, physiological role, physical pain, mental health, and vitality).
5. Nursing satisfaction. Applying our homemade nursing satisfaction questionnaire, we evaluated the satisfaction from the following five aspects: basic nursing care, nursing knowledge, nursing attitude, operation skills, and skin care, all of which are in percentage (0–100 points), and the higher the score means the more satisfied.

### 3.4 Statistical Analysis

Data analysis was performed using SPSS version 26.0 (IBM SPSS Statistics for Windows, Version 26.0.). Continuous variables were presented as mean ± standard deviation (*x¯* ± *s*) and compared between groups using independent *t* tests to determine if there were statistically significant differences in means. Categorical variables were expressed as counts and percentages (*n* [%]) and analyzed using chi‐square tests for comparison between groups. A *p* value of less than 0.05 was considered statistically significant.

## 4 Results

### 4.1 General Data Comparison

**FIGURE 1:**
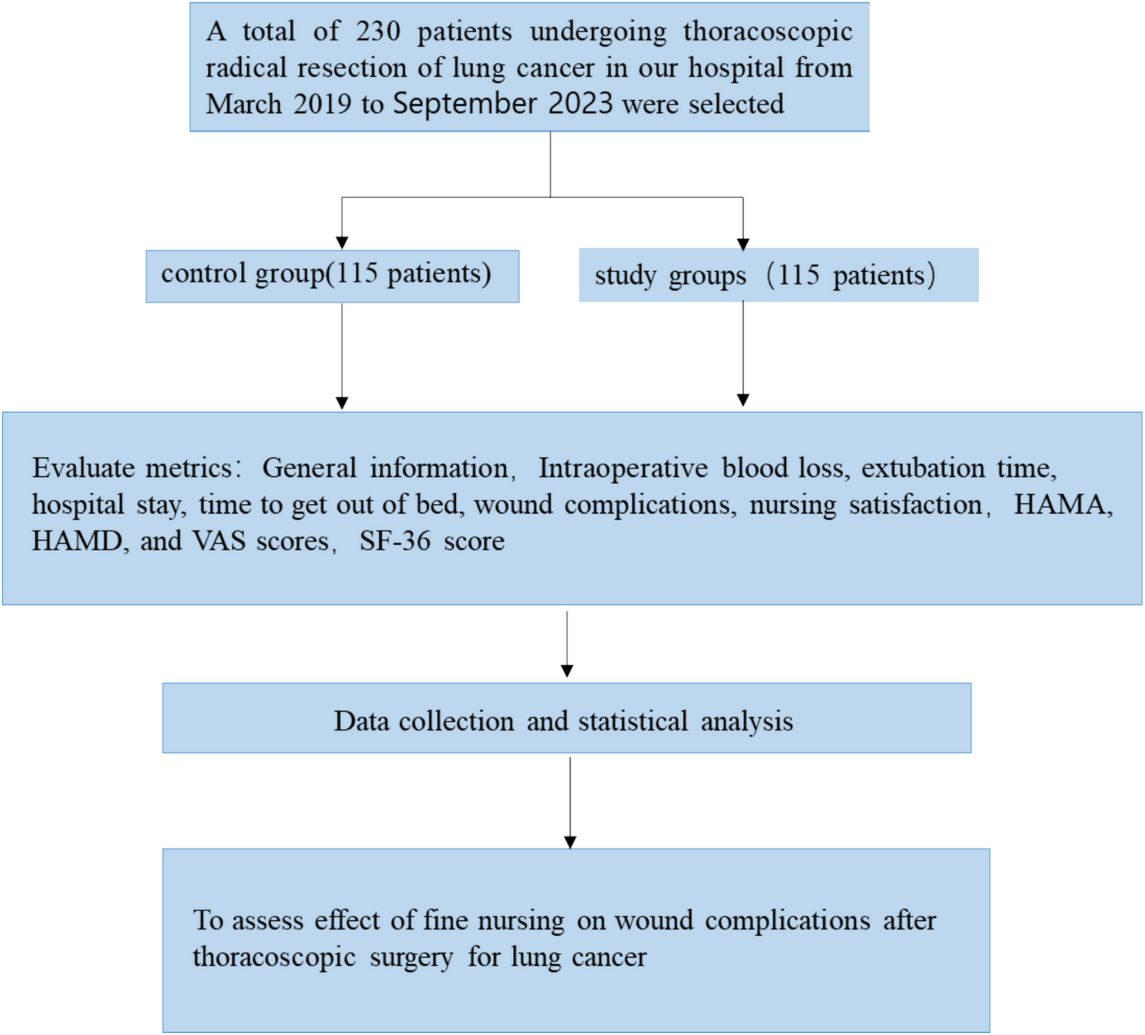
The flow chart of the study.

Flow chart for the article is shown in Figure 1.The patients' ages ranged from 53 to 71 years. A total of 65 men and 50 women were included in the control group; their average age was 58.01 ± 9.81 years; 67 urban patients and 48 rural patients; clinical stage: 98 patients in Stages I–II and 17 patients in Stage III; adenocarcinoma: 63 cases, squamous cell carcinoma: 45 cases large cell carcinoma: 4 cases, small cell carcinoma: 3 cases; 26 patients with drinking history and 89 patients with no drinking history; 43 patients with smoking history and 72 patients without smoking history; 115 patients in the study group, 63 males and 52 females; mean age 59.23 ± 9.96 years; and 69 urban patients and 46 rural patients. Clinical staging: Stages I–II 101 cases, Stage III 14 cases, adenocarcinoma: 65 cases, squamous cell carcinoma: 43 cases large cell carcinoma: 5 cases, small cell carcinoma: 2 cases; drinking history 29 cases, no drinking history 86 cases, smoking history 47 cases, nonsmoking history 68 cases. There was no significant difference between the two groups (*p* > 0.05). The difference is comparable; see Table 1.

**TABLE 1:** Comparison of general data of two groups of patients.

Index	Routine nursing group (*n* = 115)	Refined nursing group (*n* = 115)	χ2/*t*	*p*
Age (years)	58.01 ± 9.81	59.23 ± 9.96	0.826	0.426
Gender (*n*)			0.803	0.324
Male	65	63		
Female	50	52		
Clinical staging			0.974	0.283
I–II	98	101		
III	17	14		
Pathological type				
Adenocarcinoma	63	65	0.736	0.417
Squamous cell carcinoma	45	43		
Large cell carcinoma	4	5		
Small cell carcinoma	3	2		
Living environment			0.874	0.351
In the city	67	69		
In the countryside	48	46		
Drinking			0.131	0.682
Yes	26	29		
No	89	86		
Smoking history			1.358	0.279
Yes	43	47		
No	72	68		

### 4.2 Operative Indexes Comparison

**FIGURE 2:**
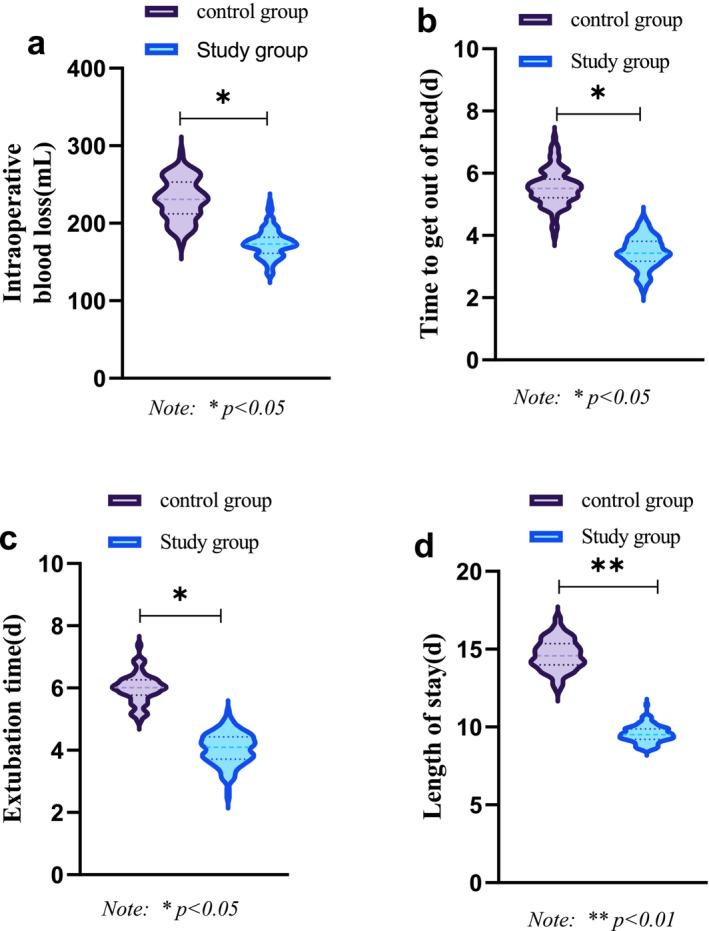
Comparison of surgical indicators between groups.

Comparing the operation‐related indicators between the two groups, Figure 2 shows the study group had less blood loss during surgery, a shorter extubation time, a shorter time to get out of bed, and a shorter hospital stay than the control group (*p* < 0.05).

### 4.3 Postoperative Wound Complications Comparison

**FIGURE 3:**
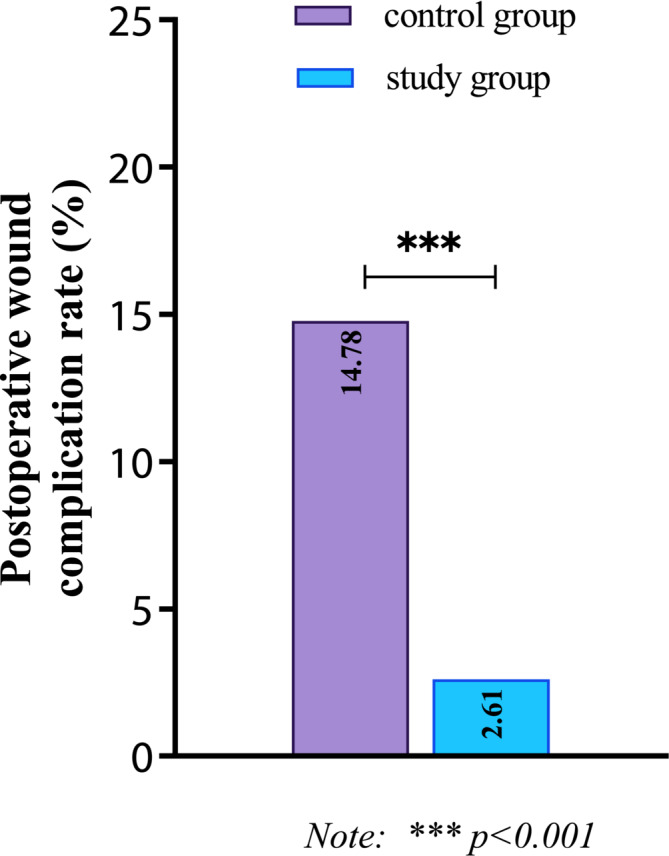
Comparison of postoperative wound complication rate between two groups after nursing intervention.

After 2 weeks of nursing intervention, there were 17 cases of wound complications in the control group, including incision infection (*n* = 5), wound bleeding (*n* = 7), pleural effusion (*n* = 3), and pulmonary air leakage (*n* = 2). Among the study group, three wound complications occurred, including one infection and two bleedings. As shown in Table 2, statistically significant (*p* < 0.05) was the difference in postoperative wound complications between the study and control groups (2.61 vs. 14.78%). See Figure 3.

**TABLE 2:** Comparison of postoperative wound complications.

Index	Routine nursing group (*n* = 115)	Refined nursing group (*n* = 115)	χ2	*p*
Incision infection	5	1	4.982	0.028
Bleeding wound	7	2		
Pleural effusion	3	0		
Pulmonary air leakage	2	0		
Total incidence of wound complications (%)	14.78	2.61		

### 4.4 HAMA, HAMD and VAS Scores Comparison

**FIGURE 4:**
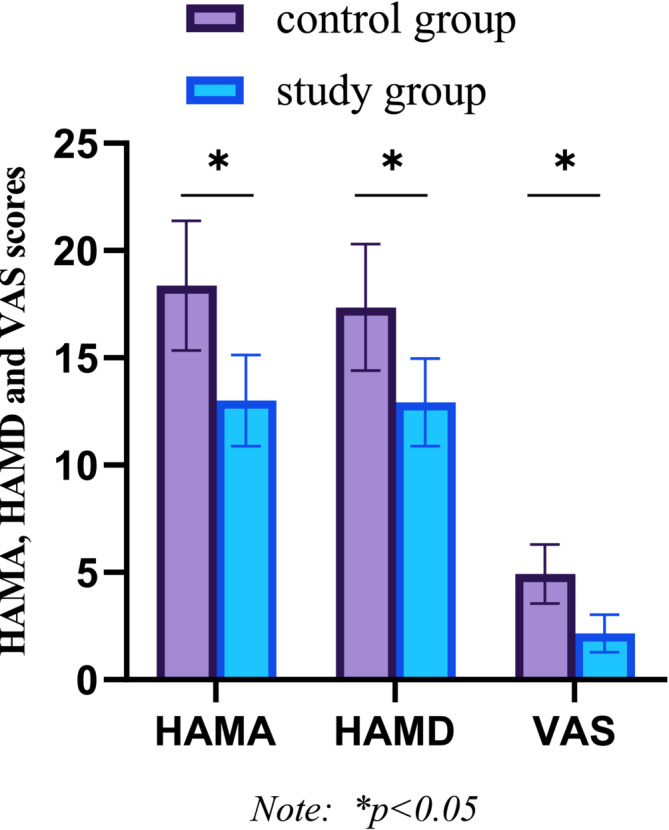
Comparison of HAMA, HAMD, and VAS scores between the two groups after nursing intervention.

Comparison of HAMA, HAMD, and VAS scores between the two groups before nursing care, the difference was not statistically significant (*p* > 0.05); after nursing care, HAMA, HAMD, and VAS scores of the study group and control group were significantly lower than those before nursing care (*p* < 0.05). See Figure 4.

### 4.5 SF‐36 Scores' Comparison

**FIGURE 5:**
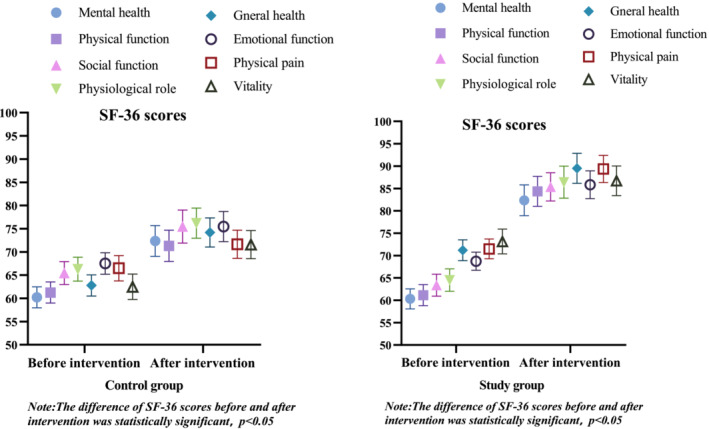
Comparison of SF‐36 scores between the two groups before and after nursing intervention.

According to Figure 5, the study group's SF‐36 scores were statistically significant (*p* < 0.05) higher than those of the control group after 2 weeks of nursing intervention.

### 4.6 Nursing Satisfaction Comparison

**FIGURE 6:**
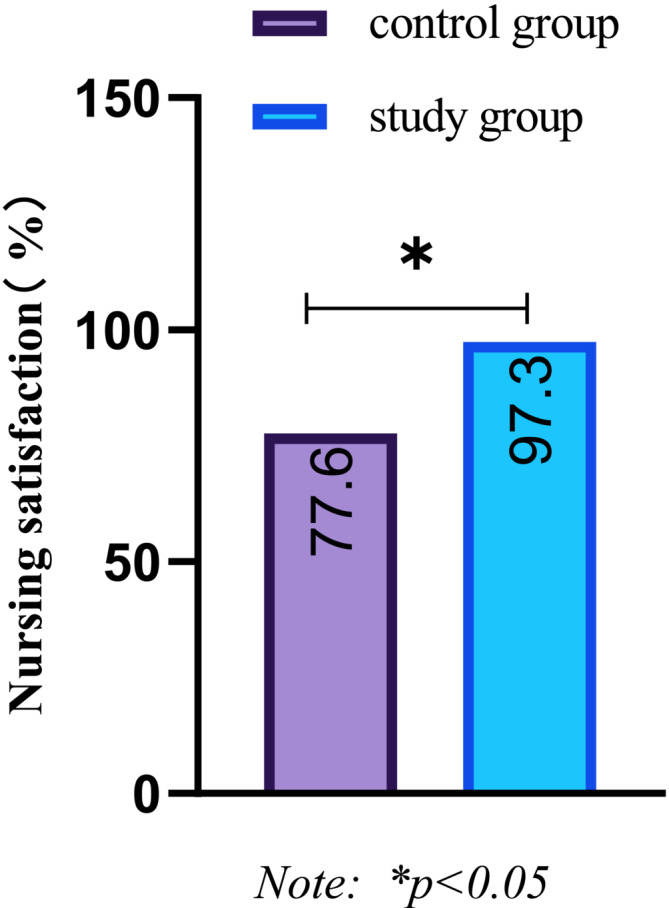
Comparison of nursing satisfaction between the two groups after nursing intervention.

After the nursing intervention, compared with the control group, the nursing satisfaction of the study group was 97.3%, which was significantly higher than the 77.6% of the control group, and the difference between the two groups was statistically significant (*p* < 0.05), as shown in Figure 6.

## 5 Discussion

At present, surgical treatment is the first choice for patients with lung cancer [18]. With the continuous progress of medical technology, thoracoscopic‐guided radical resection of lung cancer has been widely used [19]. However, some studies have found that there are higher requirements for nursing cooperation in the process of treatment, so it is necessary to implement scientific nursing cooperation at an appropriate time [20]. Refined nursing intervention links and analyzes the nursing measures, improves the nursing intervention for patients, strengthens the connection of various nursing links, and ensures the accuracy of nursing for patients [9, 21], so as to improve the effect of surgical treatment of patients and reduce postoperative wound complications.

Previous studies have shown that we should carry out refined nursing care for patients undergoing thoracoscopic radical resection of lung cancer [22, 23, 24], strengthen communication with patients before operation, and can reduce tension and anxiety. Timely communication with patients and their families after operation, observation of patients' postoperative recovery, and timely assistance to patients can reduce postoperative wound complications to a certain extent. In a meta‐analysis conducted by Wang et al. [9], the impact of operating room intensive care interventions on the incidence of surgical site wound infection in patients undergoing lung cancer surgery was evaluated. The results of the meta‐analysis showed that the intensive care intervention in the operating room significantly reduced the incidence of surgical site wound infection in patients with lung cancer surgery compared with the control group (1.82% vs. 6.52%, odds ratio: 0.30, 95% CI: 0.19–0.47, *p* < 0.001) and reduced the length of hospital stay (standardized mean difference: −1.51 days, 95% CI: −1.92 to −1.11 days, *p* < 0.001). In this study, after 2 weeks of nursing, the incidence of postoperative wound complications in the study group was 2.61%, significantly lower than 14.78% in the control group, and the difference was statistically significant (*p* < 0.05). This further confirms the results of the above research, indicating that refined nursing improves the nursing links, smoothly connects and implements various processes, and gives patients omnidirectional care, which can effectively shorten the time of operation and treatment and effectively reduce the occurrence of postoperative wound complications.

In addition, previous studies have shown that patients undergoing thoracoscopic radical resection of lung cancer have a certain degree of anxiety and depression [25, 26]. After intensive nursing intervention, the study group's scores on HAMA, HAMD, and VAS were significantly lower than those in the control group. Additionally, the study group's scores on SF‐36 were higher. This is also basically consistent with previous research results [27]. It shows that refined nursing can improve the bad mood of patients and improve their comfort. The reason may be as follows: In this study, the implementation of refined nursing can increase patients' understanding of their own condition and operation plan by communicating with patients before operation and then eliminate worries and build confidence in rehabilitation; reasonable arrangement of medical staff before operation, through careful check of patients' basic information, surgical instruments, and so on, can minimize adverse events. The implementation of intraoperative softening and postoperative guidance can improve nursing effectiveness, increase patients' satisfaction with nursing work and establish a good nurse–patient relationship.

Although this study focused on short‐term postoperative outcomes within the first 2 weeks, it is also valuable to consider the potential long‐term effects of intensive care interventions. Future studies will continue to explore outcomes such as wound healing, recurrence of complications, and overall quality of life after the immediate recovery phase. Follow‐up evaluations are conducted at 3, 6, and 12 months to understand how intensive care practices affect not only the incidence of complications but also the long‐term physical and mental health of patients.

### 5.1 Limitations

This study also has the following limitations: First, this study belongs to a single‐center retrospective study, which has an impact on the stability of the results to some extent, so further multicenter studies are needed to verify the accuracy of the results. Second, this paper discusses the effect of refined nursing intervention on wound complications after thoracoscopic radical resection of lung cancer, the time of nursing intervention is relatively short, and the long‐term effect of the two groups of nursing intervention models on patients needs to be further studied. Third, in addition to patient complications, such as hypertension and diabetes, there may be a variety of confounding factors affecting postoperative wound complications. Despite efforts to manage these variables during the analysis, it is impossible to entirely eliminate the remaining confounding factors. Determining whether the observed postoperative wound complications are caused by the interventions being studied or patient‐related factors is difficult. A future phase may involve conducting a comprehensive, prospective, multicenter randomized controlled trial to confirm the findings of this research.

## 6 Conclusion

In summary, the application of refined nursing in patients undergoing thoracoscopic radical resection of lung cancer can effectively reduce the incidence of postoperative wound complications, improve nursing satisfaction, and improve patients' quality of life.

## Author Contributions

Y.D. and G.M. designed and performed the experiments. S.W. provided support for data analysis and writing the manuscript, and S.Z. provided the supervision, resources, discussion, design, and peer review process. All the authors have seen and approved the manuscript.

## Conflicts of Interest

The authors declare no conflicts of interest.

## Data Availability

The data that support the findings of this study are available from the corresponding author upon reasonable request.
